# The complete chloroplast genome of *Aster scaber* Thunb. 1784 (Asteraceae)

**DOI:** 10.1080/23802359.2024.2447744

**Published:** 2024-12-30

**Authors:** Yue Sheng, Yu-tong Huang, Yan-ping Xing, Chun-yan Li, Zi-xin Tang, Yan-yun Yang, Liang Xu

**Affiliations:** School of Pharmacy, Liaoning University of Traditional Chinese Medicine, Dalian, China

**Keywords:** *Aster scaber*, Asteraceae, chloroplast genome, phylogeny tree

## Abstract

*Aster scaber* Thunb. (1784) is primarily distributed in eastern Asia,  has a total length of 152,778 bp and consists of a large single copy (LSC) region of 84,517 bp, a small single copy (SSC) region of 18,277 bp, and two inverted repeat (IRs) regions of 24,992 bp . The GC content is 37.31%. A total of 133 genes were annotated, including 88 protein-coding genes, 8 rRNA genes, and 37 tRNA genes. Phylogenetic analysis using the maximum likelihood method showed that *A. scaber* is closely related to *Aster* species. This study provides chloroplast genome resource for further research on the phylogenetics and resource development of *A. scaber.*

## Introduction

*Aster scaber* Thunb. (1784) is a plant from the genus *Aster* in the Asteraceae family, found in China, Korea, Japan, and eastern Siberia in Russia. It primarily grows in valleys, slopes, grasslands, and shrublands (Chen et al. 2011). Modern pharmacology has found that *A. scaber* has anti-inflammatory (Kim et al. 2022), antioxidant and lipid-lowering effects (Choi et al. 2020). Phytosterols and flavonoids from *A. scaber* have neuroprotective effects on human neuroblastoma SK-N-SH cells (Chung et al. 2016). The water extract of *A. scaber* also induces the production of nitric oxide and cytokines in lipopolysaccharids-activated macrophages (Kim et al. 2009). In addition, its seedlin, stem and leave are edible and have good nutritional value (Lei and Geng 2017).

**Figure 1.**
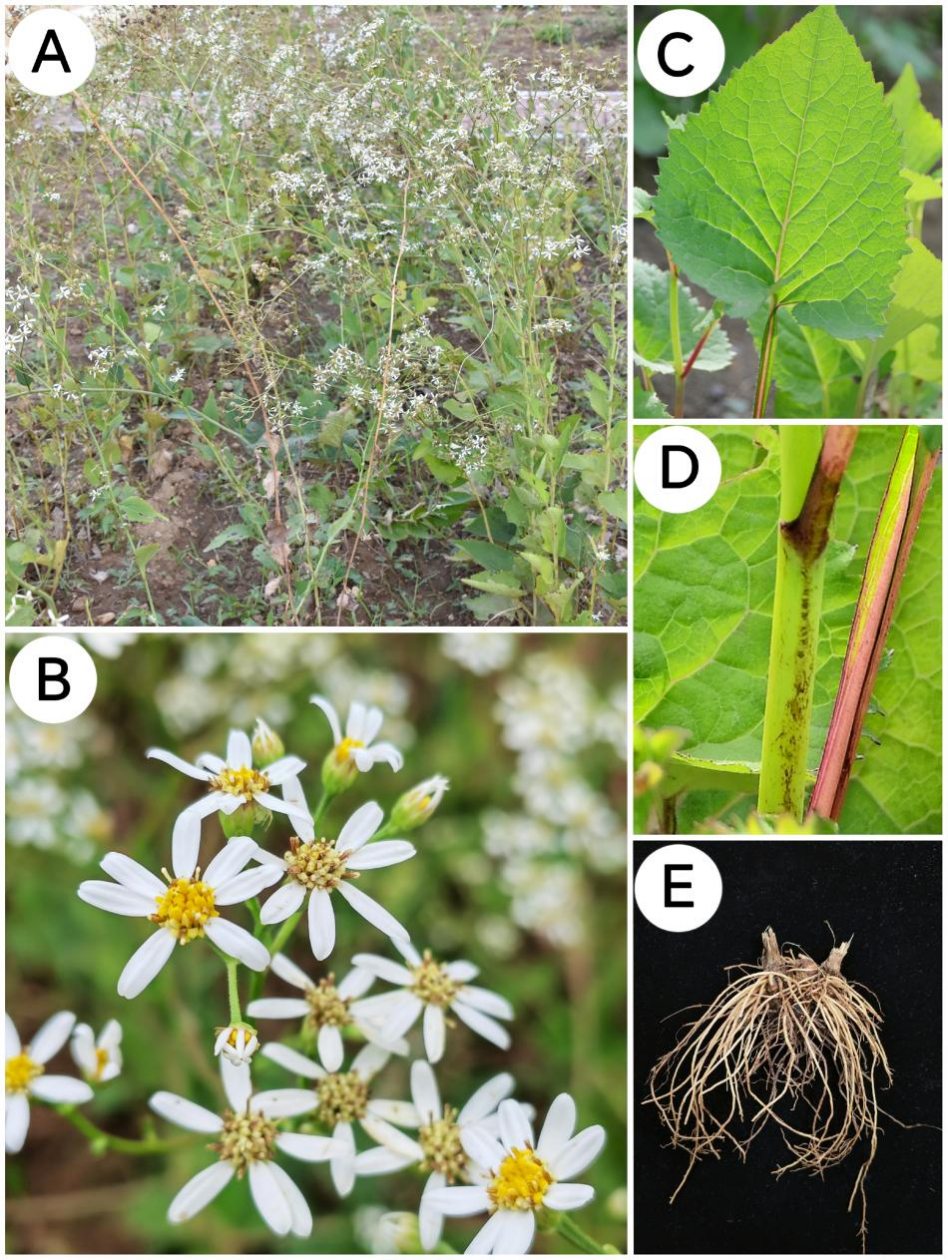
Photographs of (A) habit, (B) flower, (C) leaves, (D) stem, and (E) root and rhizome of *A. scaber* taken by Yutong Huang in Dalian, Liaoning Province, China (121°52′55.61″E, 39°3′50.40″N). Herb, perennial, fibrous root, rhizome short, thickened. Stems erect, glabrous. Leaves are reduced upward, blade ovate, base cordate, margin serrate. Upper leaves are lanceolate and sessile. Capitula are corymbiform, with white ray florets (4–9 per capitulum). Involucre campanulate. Achenes are obovoid, glabrous, and ribbed, with sordid-white, barbellate pappus bristles.

Most chloroplast genomes are closed-loop, but a variety of morphologies such as linear, D-loop configurations and lasso-like configurations also exist (Lilly et al. 2001; Bendich 2004). Plant chloroplast genomes typically consist of a large single copy (LSC) region, a small single copy (SSC) region, and a pair of inverted repeats (IRs) (Zhu et al. 2016). The length of most plant chloroplast genomes ranges from 100 to 217 kb, containing 110 to 130 genes (Kugita et al. 2003; Kim and Lee 2004). Currently, both domestic and international research on *A. scaber* primarily focuses on its pharmacological properties, with molecular studies mainly centered on chloroplast genes such as *trn*L and *psb*A (Li et al. 2012; Soejima and Hamashima 2019). However, there is a lack of comprehensive studies and analyses of the complete chloroplast genome and its genetic background. Therefore, this study combines sequencing technology and bioinformatics analysis tools to sequence and annotate the complete chloroplast genome of *A. scaber*, along with conducting a corresponding phylogenetic analysis. This will provide valuable chloroplast genome data and a theoretical basis for phylogenetic studies, DNA molecular identification, and genetic diversity research of *A. scaber* (Figure 1).

## Materials and methods

### Plant material

Fresh leaf samples were collected by Yutong Huang at the College of Pharmacy, Liaoning University of Traditional Chinese Medicine, Dalian, Liaoning Province (121°52′55.61″E, 39°3′50.40″N), and identified by Professor Liang Xu from the Liaoning University of Traditional Chinese Medicine. A specimen was deposited at the herbarium of Liaoning University of Traditional Chinese Medicine (Liang Xu 861364054@qq.com, *A. scaber* No. 10162240512001LY) (Supplementary Figure S1).

### DNA extraction and sequencing

Total genomic DNA was isolated from 150 mg of fresh leaves using the cetyltrimethylammonium bromide technique (Doyle and Doyle 1987), and DNA degradation and contamination were monitored with 1% agarose gels, while the DNA concentration was determined using the Qubit^®^ DNA Assay Kit on a Qubit^®^ 3.0 Fluorometer (Invitrogen, USA). An aliquot of 1 μg purified DNA was sonicated to fragment it into 350 bp pieces, which were used to construct a short-insert (350 bp) library with the Nextera XT DNA library preparation kit (Illumina, San Diego, CA), and the library sequencing was performed on the Illumina NovaSeq 6000 platform, with coverage assessed using the samtools depth utility.

### Genome assembly and annotation

The raw data were quality filtered using NGS QC Toolkit v2.3.3 (https://nipgr.ac.in/ngsqctoolkit.html) to obtain high-quality sequences, which were then used to assemble the complete chloroplast genome with SPAdes v3.14.1 (http://cab.spbu.ru/software/spades/) (Bankevich et al. 2012). The chloroplast genome was subsequently annotated using PGA (Qu et al. 2019), with *Aster lavandulifolius* (NC063955) as the reference genome.

### Phylogenetic analysis

To elucidate the phylogenetic position of *A. scaber* within the Asteraceae family, we retrieved the chloroplast genomes of 47 Asteraceae species from NCBI, using *Codonopsis pilosula* (NC060312) as the outgroup. The 55 shared protein-coding genes among these samples were aligned using MAFFT v7.429, and the alignments were refined with Gblocks 0.91b to remove ambiguous regions, typically those containing alignment gaps (Castresana 2000; Katoh and Standley 2013). The resulting alignments were concatenated for the construction of a phylogenetic tree. The ML tree was generated under the optimal TVM+F + R3 model, with statistical support assessed through 1000 bootstrap replicates.

## Result

### Genome structure analysis

**Figure 2.**
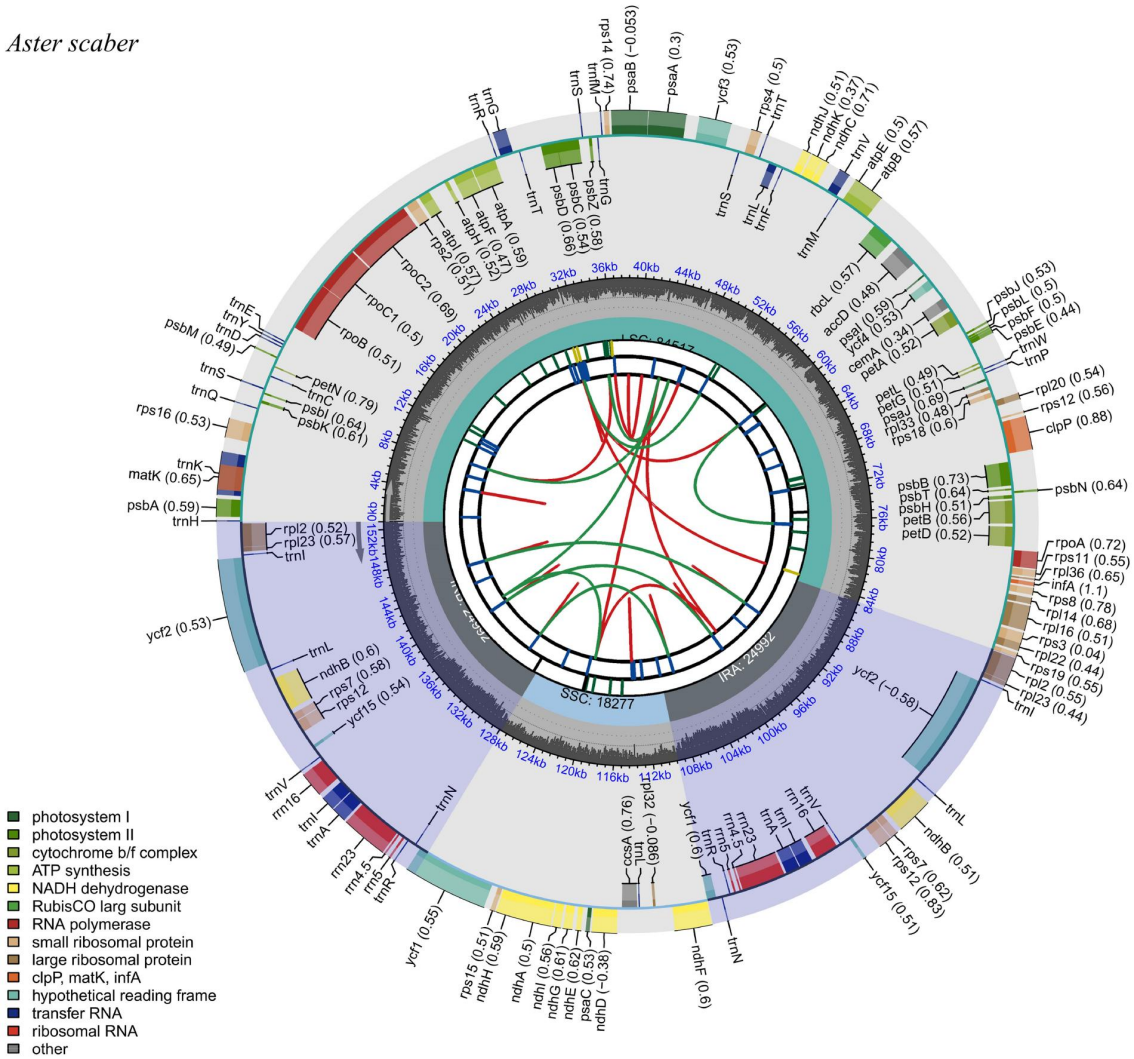
Schematic map of overall features of *A. scaber* chloroplast genome. The species name is shown in the left top corner. The map contains six tracks in default. From the center outward, the first track shows the dispersed repeats. The dispersed repeats consist of direct (D) and palindromic (P) repeats, connected with red and green arcs. The second track shows the long tandem repeats as short blue bars. The third track shows the short tandem repeats or microsatellite sequences as short bars with different colors. The SSC, IRs (IRa and IRb), and LSC regions are shown on the fourth track. The GC content along the genome is plotted on the fifth track. The base frequency at each site along the genome will be shown between the fourth and fifth tracks. The genes are shown on the sixth track. The optional codon usage bias is displayed in the parenthesis after the gene name. Genes are color-coded by their functional classification. The transcription directions for the inner and outer genes are clockwise and anticlockwise, respectively. The functional classification of the genes is shown in the bottom left corner.

The sequencing of the *A. scaber* chloroplast genome yielded a total of 4.51 GB of raw data, with 4.45 GB of clean data remaining after filtering. Of this, 98.11% of the data had a quality score above Q20. The assembled and annotated chloroplast genome was 152,778 bp in length, with the average sequencing depth of 1336.03×, the maximum depth of 7997×, and the minimum depth of 468× (Supplementary Figure S2). A total of 133 genes were identified, including 88 protein-coding genes, 8 rRNA genes, and 37 tRNA genes. Several genes, such as *atp*F, *ndh*A, *ndh*B, *pet*B, *pet*D, *rpl*16, *rpl*2, *rpo*C1, *rps*16, *trn*A-UGC, *trn*G-UCC, *trn*I-GAU, *trn*K-UUU, *trn*L-UAA, and *trn*V-UAC, contained a single intron, while *clp*P and *ycf*3 had two introns each. Additionally, the *rps*12 gene exhibited trans-splicing. The annotated chloroplast genome map, along with the cis-splicing and trans-splicing gene maps (Figure 2, Supplementary Figures S3 and S4, respectively) for *A. scaber*, were generated using the PAG tool (Qu et al. 2019) and visualized with CPGview (Liu et al. 2023).

**Figure 3.**
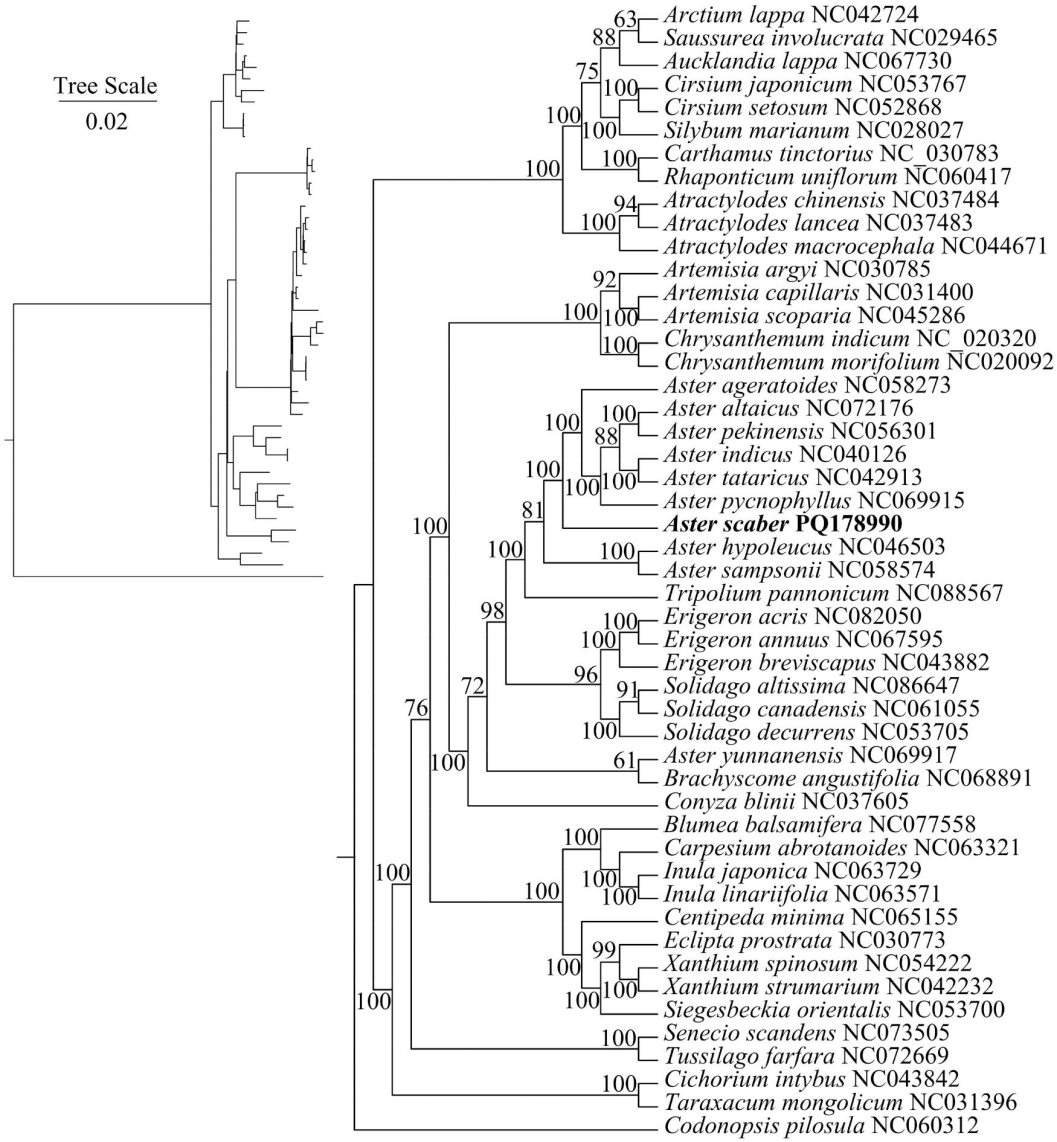
Maximum likelihood (ML) phylogenetic tree for *A. scaber*. Whole genome sequence of *A. scaber* and 48 other chloroplasts. Numbers above branches indicate bootstrap values for ML analysis. The best evolutionary model was selected as TVM+F + R3, which was chosen using ModelFinder. The scale bar in the lower left corner of the figure represents the evolutionary distance in units of length 0.02. The following sequences were used: *Aster hypoleucus* NC046503 (Wang et al. 2019), *Aster indicus* NC040126 (Liu et al. 2018), *Aster pekinensis* NC056301 (Zhang et al. 2021), *Aster ageratoides* NC058273, *A. tataricus* NC042913 (Shen et al. 2018), *Aster altaicus* NC072176, *A. pycnophyllus* NC069915, *Aster sampsonii* NC058574 (Chang et al. 2021), *Aster yunnanensis* NC069917, *Erigeron breviscapus* NC043882, *Erigeron annuus* NC067595, *Erigeron acris* NC082050, *Solidago canadensis* NC061055, *Solidago decurrens* NC053705, *Solidago altissima* NC086647, *brachyscome angustifolia* NC068891, *Tripolium pannonicum* NC088567, *Xanthium strumarium* NC042232, *Xanthium spinosum* NC054222, *Artemisia argyi* NC030785, *Artemisia scoparia* NC045286(Iram et al. 2019), *Artemisia scoparia* NC031400 (Lee et al. 2016), *Atractylodes lancea* NC037483, *Atractylodes chinensis* NC037484, *Atractylodes macrocephala* NC044671, *Aucklandia lappa* NC067730, *Blumea balsamifera* NC077558 (Zhao et al. 2021), *Carpesium abrotanoides* NC063321, *Carthamus tinctorius* NC030783, *Centipeda minima* NC06515, *Chrysanthemum indicum* NC020320, *Chrysanthemum morifolium* NC020092, *Cichorium intybus* NC043842, *Cirsium japonicum* NC053767, *Cirsium setosum* NC052868 (Xie et al. 2019), *Conyza blini* NC037605, *Eclipta prostrata* NC030773 (Park et al. 2016), *Inula japonica* NC063729, *Inula linariifolia* NC063571, *Rhaponticum uniflorum* NC060417, *Saussurea involucrata* NC029465 (Xie et al. 2017), *Senecio scandens* NC073505, *Siegesbeckia orientalis* NC053700 (Liu et al. 2019), *Silybum marianum* NC028027, *Taraxacum mongolicum* NC031396 (Kim et al. 2016), *Tussilago farfara* NC072669, *Arctium lappa* NC042724, *C. pilosula* NC060312.

### Phylogenetic analysis

The topology of phylogenetic tree revealed evolutionary relationships between *A. scaber* and the other 48 species, supported by high bootstrap values. Phylogenetic tree analysis revealed tha*t A. scaber* is closely related to *Aster* species such as *A. pycnophyllus* and *A. tataricus*, clustering together into one clade. The establishment of the phylogenetic tree will be helpful for further study of the *A. scaber* (Figure 3).

## Conclusion and discussion

This study presents the first annotation and report of the *A. scaber* chloroplast genome. Structurally, it exhibits the typical quadripartite arrangement found in most plant chloroplasts, consisting of LSC, SSC, and IRs (Zhu et al. 2016). *Aster* is a large genus in the Asteraceae family. In recent years, with advancements in sequencing technology, numerous studies on gene annotation and classification of *Aster* have been conducted. Such as phylogenetic relationships between *Aster* and related species have been analyzed using both nuclear and chloroplast gene data (Li et al. 2012). This study also analyzed the phylogenetic relationships among *A. scaber*, *Aster* species, and other genera using shared protein-coding genes in the chloroplast genome. The results showed that *A. scaber* and *Aster* species clustered together into a single branch, and most of other *Aster* species also clustered together in a single branch. Similar clustering results have been observed in studies of other *Aster* species, such as *A. ageratoides*, *A. sampsonii*, *A. altaicu* and *A. souliei*, using phylogenetic trees constructed from shared protein-coding genes in the chloroplast genome (Feng et al. 2021; Wang et al. 2022; Wang and Liu 2023). These findings further demonstrate that chloroplast genome analysis effectively differentiates the phylogenetic relationships among *Aster* species.

The findings of this study not only provide valuable insights into the phylogenetic evolution of *A. scaber* but also offer important chloroplast genome data that can aid in species identification and resource development of *A. scaber.* Moreover, research on the chloroplast genome of *A. scaber* has been relatively scarce, with most studies focusing only on partial chloroplast genes such as *trn*L and *psb*A (Li et al. 2012; Soejima and Hamashima 2019), lacking comprehensive, systematic research. The complete sequencing of the *A. scaber* chloroplast genome reported in this study will stimulate further research in this area and could potentially lead to the discovery of more medicinal values based on the full chloroplast genome information.

## Supplementary Material

Supplementary materials.docx

## Data Availability

The genome sequence data that support the findings of this study are openly available in GenBank of NCBI at (https://www.ncbi.nlm.nih.gov/) under accession no. PQ178990. The relevant BioProject, SRA, and BioSample numbers are PRJNA1140890, SRR30012505 (Illumina), and SAMN42866185, respectively.
