# Supplementary material for: The complete chloroplast genome of Aster scaber Thunb. 1784 (Asteraceae)
Source: Mitochondrial DNA B Resour. 2024 Dec 30;10(1):57–62. doi: 10.1080/23802359.2024.2447744 (PMC11703284; doi:10.1080/23802359.2024.2447744)
Supplement: Supplementary materials.docx [file TMDN_A_2447744_SM9564.docx]

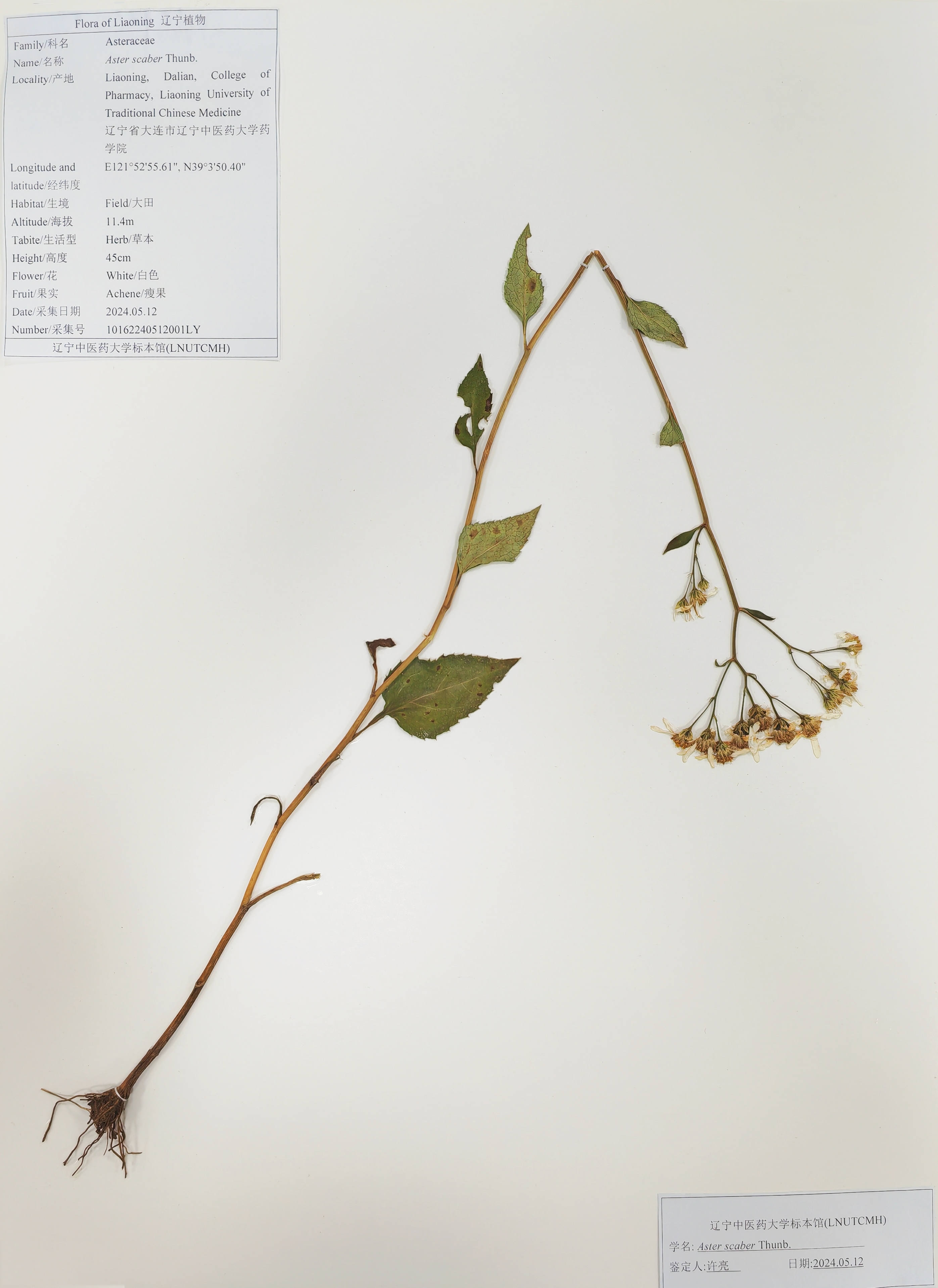


**Figure S1.** An illustration of *Aster scaber* Thunb. specimen preserved at Liaoning University of Traditional Chinese Medicine. Specimen number, collector, latitude and longitude and other details are noted on the figure for reference.


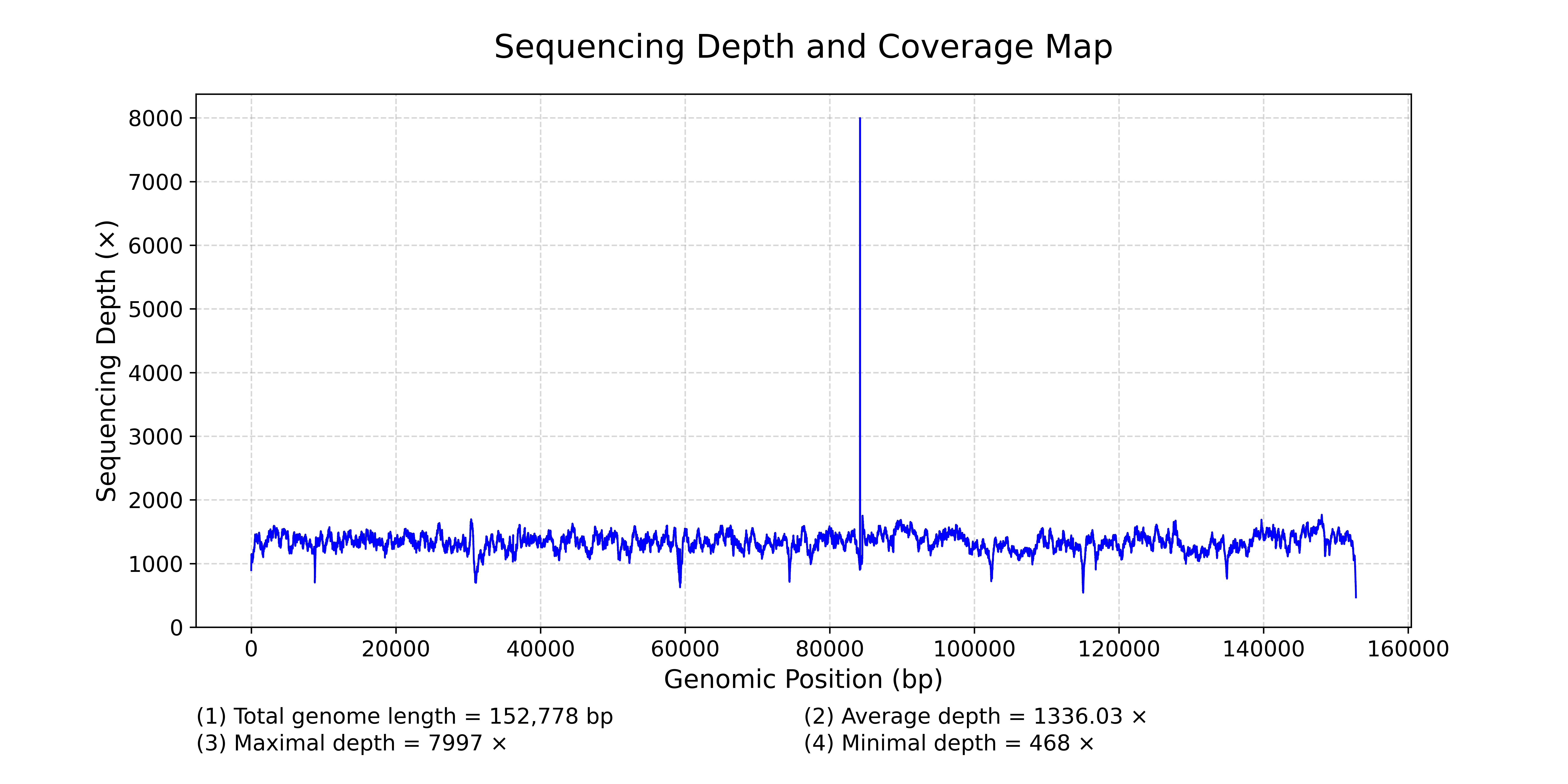


**Figure S2.** Sequencing coverage depth of *Aster scaber* Thunb. The illumina short sequences were compared to the chloroplast genome sequences using BWA software and finally the coverage was calculated using samtools depth (The maximum sequencing depth was 7997 ×, the minimum sequencing depth was 468 ×, and the average sequencing depth was 1336.03 ×). The horizontal coordinate is the chloroplast length and the vertical coordinate is the coverage depth.

Li H. 2013. Aligning sequence reads, clone sequences and assembly contigs with BWA-MEM. arXiv Prepr arXiv. 0(0):3.

Li H. et al. 2009. The Sequence Alignment/Map format and SAMtools. Bioinformatics, 25, 2078-2079.


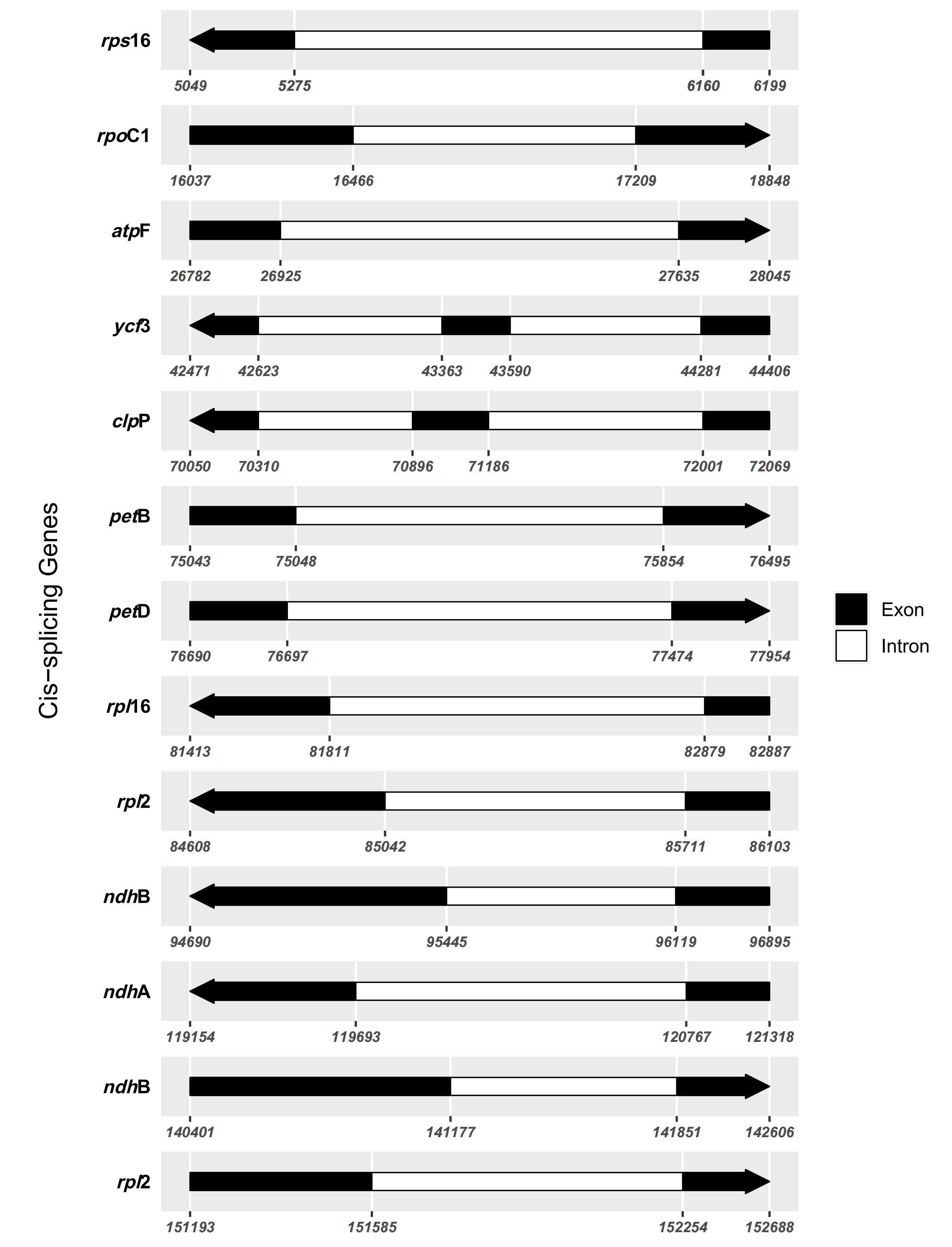


**Figure S3.** Schematic map of the cis-splicing genes in the chloroplast genome. The genes are arranged from top to bottom based on their order on the chloroplast genome. The gene names are shown on the left, and the gene structures are on the right. The exons are shown in black; the introns are shown in white. The arrow indicates the sense direction of the gene. Please note that lengths of exons and introns are not drawn to scale.


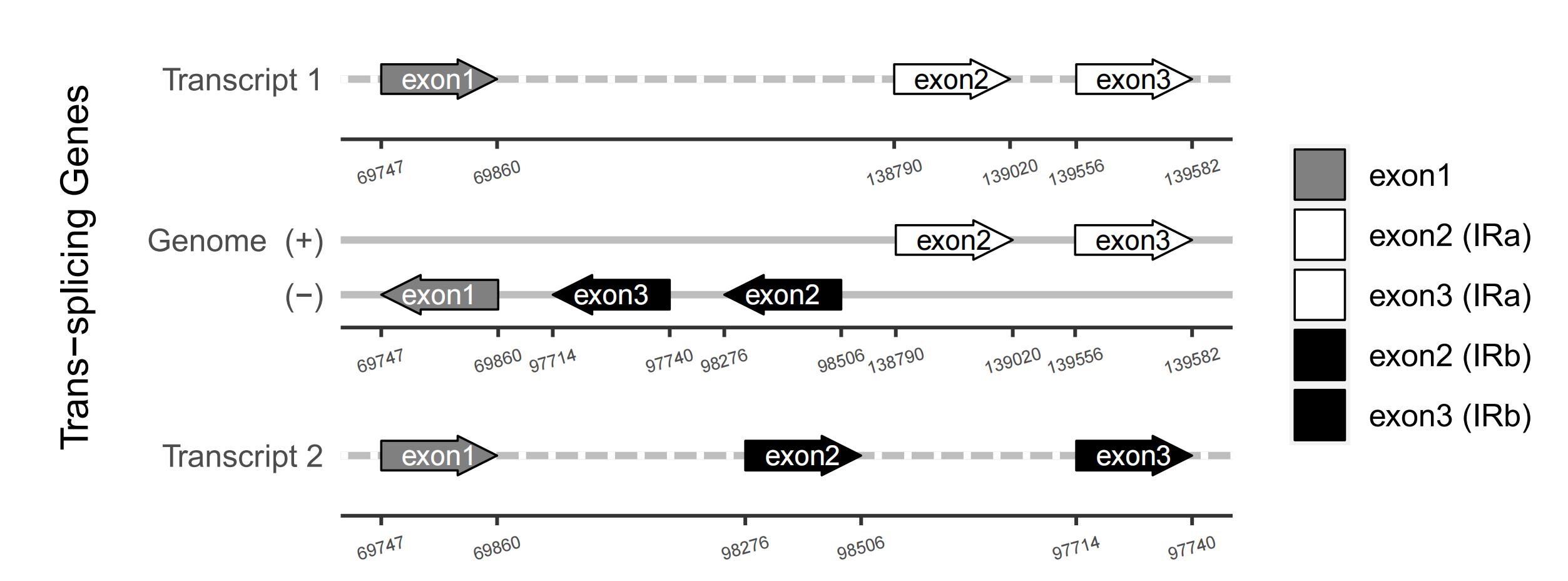


**Figure S4.** Schematic map of the trans-splicing gene *rps*12 in the chloroplast genome. It has three unique exons. Two of them are duplicated as they are located in the IR regions.
